# Recanalization of rectal anastomosis atresia with magnetic compression anastomosis

**DOI:** 10.1055/a-2432-2279

**Published:** 2024-10-25

**Authors:** Miaomiao Zhang, Huanchen Sha, Hairong Xue, Yicong Li, Yun Li, Yi Lv, Xiaopeng Yan

**Affiliations:** 1 162798 Department of Hepatobiliary Surgery, The First Affiliated Hospital of Xiʼan Jiaotong University, Xiʼan, China; 2 162798 Shaanxi Provincial Key Laboratory of Magnetic Medicine, The First Affiliated Hospital of Xiʼan Jiaotong University, Xiʼan, China; 3 162798 Department of Gastroenterology, The First Affiliated Hospital of Xiʼan Jiaotong University, Xiʼan, China

A 71-year-old man underwent radical resection of rectal cancer and ileostomy 11 months prior. Two weeks ago, an ileostomy closure was planned; however, a colonoscopy revealed atresia of the rectal anastomosis. The patient came to our hospital seeking a magnetic surgical treatment technique. Following colonoscopy and colonography, which clearly demonstrated rectal anastomotic atresia ( Fig. 1 ), we developed a detailed treatment plan of magnetic compression anastomosis ( Fig. 2 ).

**Fig. 1:**
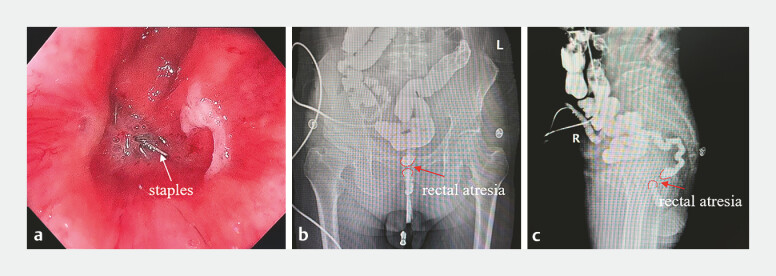
The atresia of the rectum. **a** Colonoscopy. **b, c** Colonography.

**Fig. 2:**
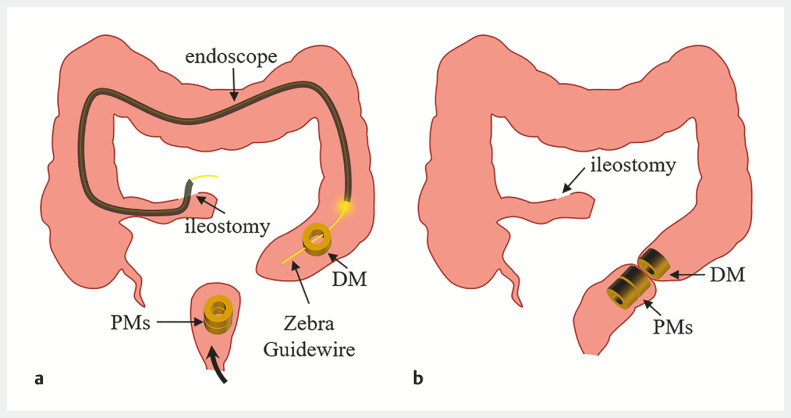
Surgical planning. **a** The daughter magnet (DM) and the parent magnets (PMs) were inserted through the ileostomy and anus, respectively. **b** The daughter magnet and parent magnets attracted together.

The procedure was performed after the patient and his family signed the informed consent form. Following anesthesia, an endoscope was inserted into the colon through the ileostomy; however, reaching the proximal end of the atresia proved challenging. Consequently, a Zebra guidewire was introduced through the biopsy hole to navigate as close as possible to the proximal end of the atresia. The guidewire was maintained in position while the colonoscope was withdrawn. The tail end of the guidewire passed through the side hole of the daughter magnet, which was then advanced along the guidewire using the endoscope to approach the proximal end of the atresia as closely as possible. Subsequently, the guidewire and endoscope were removed ( Fig. 3 ). After the daughter magnet reached the proximal end of the rectal atresia, the parent magnets were inserted through the anus to the distal end of the atresia, where the parent magnets and daughter magnet were attracted together ( Fig. 4 , Video 1 ).

**Fig. 3:**
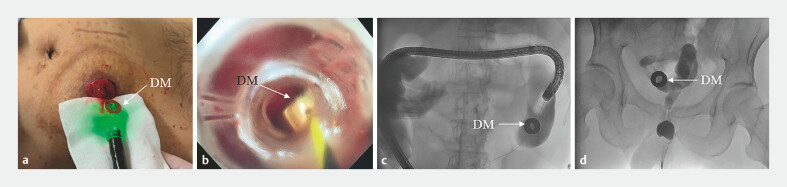
Insertion of the daughter magnet. **a** The guidewire passed through the side hole of the daughter magnet. **b** The daughter magnet was pushed towards the proximal end of the atresia by endoscope. **c** The status of the daughter magnet observed by X-ray. **d** The daughter magnet was pushed as proximal to the atresia as possible.

**Fig. 4:**
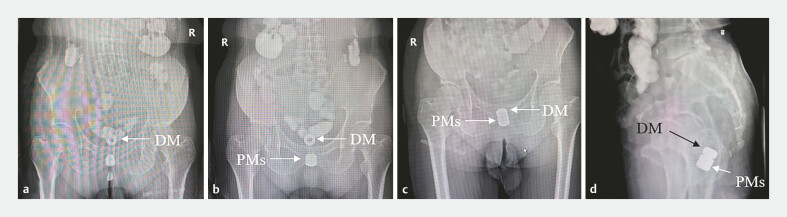
Placement of the parent magnets. **a** The daughter magnet reached the proximal end of the atresia on its own. **b** The parent magnets were inserted through anus. **c, d** Anteroposterior and lateral view after the parent magnets and daughter magnet attracted together.

The surgical procedure of magnetic compression anastomosis.Video 1

After 14 days, the magnets were removed via the anus. A three-month follow-up revealed that the anastomotic site was patent ( Fig. 5 ).

**Fig. 5:**
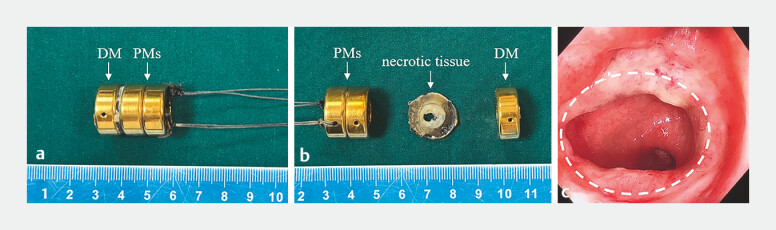
Establishment of the anastomosis. **a** The magnets were removed 14 days after surgery. **b** The compressed necrotic tissue between the daughter magnet and parent magnets can be seen after separating them. **c** Three months later, a colonoscopy showed that the anastomosis was still unobstructed.

Magnetic compression anastomosis has previously been used for the recanalization of colorectal and esophageal obstructions 1 2 3 4 . The successful recanalization of atresia in this case highlights the benefits of combining magnetic compression anastomosis with endoscopy for the treatment of a gastrointestinal obstruction.

Endoscopy_UCTN_Code_TTT_1AQ_2AF

## Notes

Endoscopy E-Videos https://eref.thieme.de/e-videos
*E-Videos* is an open access online section of the journal *Endoscopy* , reporting on interesting cases and new techniques in gastroenterological endoscopy. All papers include a high-quality video and are published with a Creative Commons CC-BY license. Endoscopy E-Videos qualify for HINARI discounts and waivers and eligibility is automatically checked during the submission process. We grant 100% waivers to articles whose corresponding authors are based in Group A countries and 50% waivers to those who are based in Group B countries as classified by Research4Life (see: https://www.research4life.org/access/eligibility/ ). This section has its own submission website at https://mc.manuscriptcentral.com/e-videos .
